# Pneumococcal vaccination coverages by age, sex and specific underlying risk conditions among middle-aged and older adults in Catalonia, Spain, 2017

**DOI:** 10.2807/1560-7917.ES.2019.24.29.1800446

**Published:** 2019-07-18

**Authors:** Angel Vila-Córcoles, Olga Ochoa-Gondar, Cinta de Diego, Eva Satué, Angel Vila-Rovira, Maria Aragón

**Affiliations:** 1 Primary Health Care Service Camp de Tarragona, Tarragona, Spain; 2 Unitat de Suport a la Recerca of Tarragona, Institut Universitari d'Investigacio en Atencio Primaria Jordi Gol (IDIAP Jordi Gol), Tarragona, Spain; 3 Primary Care Research Institute Jordi Gol. Barcelona, Spain; 4 Information System for the Improvement of Research in Primary Care (SIDIAP), Primary Care Research Institute Jordi Gol, Universitat Autonoma de Barcelona, Barcelona, Spain

**Keywords:** adults, coverage, pneumococcal conjugate vaccine, pneumococcal polysaccharide vaccine, pneumococcal vaccination

## Abstract

**Background:**

Recent published data on pneumococcal vaccination coverages among adults are scarce.

**Aim:**

To update on pneumococcal vaccination uptakes among middle-aged and older adults in Catalonia.

**Methods:**

We conducted a population-based retrospective observational study including 2,057,656 individuals ≥ 50 years old assigned to primary care centres managed by the Catalonian Health Institute on 1 January 2017 (date of data collection). An institutional clinical research database (SIDIAP) was used to classify persons by vaccination status for both 23-valent pneumococcal polysaccharide (PPsV23) and 13-valent pneumococcal conjugate (PCV13) vaccines, as well as to identify underlying risk conditions.

**Results:**

Overall, 796,879 individuals (38.7%) had received PPsV23 and 13,607 (0.7%) PCV13. PPsV23 coverage increased with age: 9.2% (95,409/1,039,872) in 50–64 year olds, 63.1% (434,408/688,786) in 65–79 year olds and 81.2% (267,062/328,998) in ≥ 80 year olds (p < 0.001). PCV13 coverage also increased with age, although percentages were smaller in all age strata (4,250/1,039,872: 0.4%; 6,005/688,786: 0.9% and 3,352/328,998: 1.0%, respectively; p < 0.001). By sex, no substantial coverage differences were observed. Considering publically funded target groups for PPsV23 vaccination in Catalonia (i.e. < 65 year olds with at least one risk factor, plus all adults aged ≥ 65 years), PPsV23 coverage reached 52.8% (771,722/1,462,261) in our study population. Regarding PCV13 publicly funded targets (i.e. all-age immunocompromised persons), PCV13 coverage was 3.3% (6,617/202,348). By risk conditions, the highest PPsV23 coverage appeared in congestive heart failure (51,909/63,596; 81.6%), chronic renal disease (122,791/158,726; 77.4%) and chronic bronchitis/emphysema (96,453/132,306; 72.9%). Maximum PCV13 coverage appeared in cirrhosis (294/7,957; 3.7%), chronic renal disease (5,633/158,726; 3.5%) and chronic bronchitis/emphysema (2,859/132,306; 2.2%).

**Conclusion:**

Pneumococcal vaccination coverages in Catalonian adults are suboptimal, especially for PCV13.

## Introduction

Infections caused by *Streptococcus pneumoniae* are an important cause of morbidity and mortality around the world [1]. At present, two pneumococcal vaccines, the classical 23-valent pneumococcal polysaccharide vaccine (PPsV23) and the new 13-valent pneumococcal conjugate vaccine (PCV13), are available for using in at-risk individuals and older adults [2,3].

In the United States (US), a leading setting on pneumococcal vaccination in adults, the Healthy People 2020 goals include to increase pneumococcal vaccination coverages to 60% in high-risk adults aged 18–64 years and to 90% in all people aged ≥ 65 years [4]. However, few recent coverage data (i.e. after PCV13 approval for adults in 2012) are available [5-7]. Updated data on both PPsV23 and PCV13 vaccination coverages in adults are therefore needed, especially now that pneumococcal vaccination programmes for adults and children are routinely implemented in many settings. Moreover, these data are also relevant to evaluate direct and indirect protective effects (e.g. herd protection) on the population [8].

In Catalonia, similar to the rest of Spain, PPsV23 is publicly funded since the 2000s for all individuals aged ≥ 65 years (with or without risk conditions) and for individuals 18–64 years old with certain at-risk conditions (i.e. immunocompromising conditions, chronic pulmonary/respiratory diseases, heart diseases, chronic renal disease, diabetes mellitus, cirrhosis, alcoholism and/or smoking). PPsV23 revaccination is recommended and publicly funded for those persons who received a prime dose before 65 years of age [9]. Since 2012 the PCV13 is publicly funded only for some individuals at high risk (mainly immunocompromised persons). The PCV13 is also prescribed by some clinicians for patients with certain at-risk conditions (i.e. chronic bronchitis, asthma, cardiac diseases, etc.) although it is not publicly funded for these patients [9,10].

This study reports current pneumococcal vaccination uptakes for both PPsV23 and PCV13 vaccines among the general adult population ≥ 50 years old in Catalonia in January 2017, assessing vaccine coverages by age, sex and presence of major underlying risk conditions.

## Methods

### Study type

This is a population-based retrospective observational study involving 2,057,656 individuals aged ≥ 50 years, who constituted all persons registered in the primary care centres (PCCs) managed by the Catalonian Health Institute (ICS, Institut Català de la Salut) in Catalonia on 1 January 2017 (date of the study).

### Ethical statement

The study was approved by the ethical committee of the institution (file P14/134) and was conducted in accordance with the general principles for observational studies [11].

### Setting and study population

In Catalonia, similar to the rest of Spain, a compulsory health insurance system by the National Health Service covers all inhabitants’ medical needs and all inhabitants are assigned to a PCC. In Catalonia there are 358 PCCs, including 274 (76.5%) managed by the ICS and 84 by other providers. The study population assigned to the 274 PCCs managed by the ICS (n = 2,057,656 persons aged ≥ 50 years) represents a 76.3% of the total 2,693,570 Catalonian inhabitants in this age strata [12].

In all PCCs, doctors and nurses systematically use electronic medical records to record diagnoses, prescriptions, vaccinations and patient management activities coded according to the International Classification of Diseases, 10th Revision (ICD-10) [13].

### Data sources

The Catalonian Health Institute Information System for the Development of Research in Primary Care (SIDIAP research database), which compiles coded clinical information from the electronic medical records of the 274 PCCs managed by the ICS, was used as main data source for this report [14]. The SIDIAP sample is representative of the general Catalan population in terms of geographical, age and sex distributions according to census data [12], and its utility for clinical research has been previously reported [15]. In 2012, the US Food and Drug Administration (FDA) and the European Medicines Agency (EMA) approved PCV13 use in adults  ≥ 50 years old [16,17]. Subsequently, information on pneumococcal vaccination with this vaccine for this age group was recorded in the SIDIAP research database. Vaccination with PPsv23 is recorded since 1999.

The SIDIAP research database was used to classify study persons by their pneumococcal vaccination status as well as to identify comorbidities and underlying risk conditions in each individual. The following risk conditions were defined on the basis of ICD-10 codes registered in the electronic PCCs medical records contained in the SIDIAP research database: asthma (J45–J46), chronic bronchitis/emphysema (J41–J44), chronic renal disease (N18–N19), congestive heart failure (I50), coronary artery disease (I20–I22, I25), diabetes mellitus (E10–E14), liver cirrhosis (K74), solid organ or haematological neoplasia (C00–C97), as well as alcoholism (F10, G31.2, G62.1, G72.1, I42.6, K29.2, K70) and current smoking (F17). We considered healthy (immunocompetent persons without risk conditions), at-risk (immunocompetent persons with any risk condition) and high-risk (immunocompromised persons) groups. Immunocompromisation was considered as a composite variable defined by the presence of any of the following: HIV infection, immunodeficiency, severe renal disease, solid organ or haematological neoplasia and/or immunosuppressive treatment.

### Review of vaccination status

Pneumococcal vaccination status for PPsV23 and PCV13 was determined by reviewing the electronic PCCs clinical records of the SIDIAP database, which contain specially designated fields for both PCV13 and PPsV23 pneumococcal vaccinations (virtually all of them are administered at the PCCs in the Spanish healthcare system). We assumed that information in electronic clinical records was complete, so a person was considered as unvaccinated when a vaccination was not recorded. Persons were considered vaccinated against pneumococcus if they had received at least one dose of PPsV23 or PCV13.

### Data analysis

Vaccination coverage for both PPsV23 and PCV13 was estimated by age strata, sex and presence of at-risk conditions. The chi-squared test was used to compare proportions of PPSV23/PCV13 vaccinated vs non-vaccinated in the initial univariate analyses. Unadjusted odds ratios (ORs) were calculated to estimate the probability of vaccination according to each one of the studied covariables (age strata, sex and presence of distinct risk conditions). We did logistic regression analyses (with the ‘enter’ method including all covariables with a significance level p < 0.10 in previous univariate analyses) to calculate multivariable-adjusted ORs for vaccination (PPSV23 and/or PCV13) [18]. Statistical significance was set at p < 0.05 (two-tailed). Data were analysed by using IBM SPSS Statistics for Windows, version 24 (IBM Corp., Armonk, New York, US).

## Results

The studied population included 2,057,656 persons, of whom 950,042 (46.2%) were men and 1,107,614 (53.8%) were women. By age strata, 1,039,872 (50.5%) were aged 50–64 years, 688,786 (33.5%) were aged 65–79 years and 328,998 (16.0%) were aged ≥ 80 years.

Considering chronic respiratory diseases, 132,306 study persons (6.4%) had chronic bronchitis/emphysema and 95,600 (4.6%) had asthma. Considering other at-risk conditions, 350,511 study persons (17.0%) had diabetes mellitus, 344,471 (16.7%) were current smokers, 236,623 (11.5%) had cancer, 158,726 (7.7%) chronic renal disease, 117,575 (5.7%) coronary artery disease, 63,596 (3.1%) congestive heart failure, 64,829 (3.2%) abused alcohol and 7,957 (0.4%) had cirrhosis.

Overall, 796,879 persons (38.7%) had received PPsV23 at any time (186,242 vaccinated within the last 5 years) and 13,607 (0.7%) had received PCV13 (all of them within the previous 5 years). Of the 796,879 PPsV23 vaccinated persons, 121,595 had received two doses and 2,347 received three or more doses. Of the 13,607 PCV13 vaccinated persons, 148 had received two doses and 60 received three doses. Table 1 shows both PPSV23 and PCV13 pneumococcal vaccination coverages by sex and age strata. PPsV23 coverage largely increased with increasing age (9.2% in 50–64 years vs 63.1% in 65–79 years vs 81.2% in ≥ 80 years; p < 0.001) and was slightly lower in men than in women (37.4% vs 39.9%; p < 0.001). PCV13 coverage also increased with age, although it was very low in all age groups (0.4% in 50–64 years vs 0.9% in 65–79 years vs 1.0% in ≥ 80 years; p < 0.001) and was slightly greater in men than in women (0.8% vs 0.6%; p < 0.001).

**Table 1:** Pneumococal vaccination coverages with PPsV23 and PCV13 by sex and age strata, Catalonia, Spain, 2017 (n = 2,057,656)

Characteristic	PPsV23		PCV13	
	n	%	n	%
**Aged 50–64** **years**				
Men (n = 512,108)	51,120	10	2,409	0.5
Women (n = 527,764)	44,289	8.4	1,841	0.3
All (n = 1,039,872)	95,409	9.2	4,250	0.4
**Aged 65–79** **years**				
Men (n = 316,831)	203,020	64.1	3,430	1.1
Women (n = 371,955)	231,388	62.2	2,575	0.7
All (n = 688,786)	434,408	63.1	6,005	0.9
**Aged ≥ 80** **years**				
Men (n = 121,103)	101,199	83.6	1,637	1.4
Women (n = 207,895)	165,863	79.8	1,715	0.8
All (n = 328,998)	267,062	81.2	3,352	1.0
**All-age groups**				
Men (n = 950,042)	355,339	37.4	7,476	0.8
Women (n = 1,107,614)	441,540	39.9	6,131	0.6
Overall (n = 2,057,656)	796,879	38.7	13,607	0.7

PPsV23: 23-valent pneumococcal polysaccharide vaccine; PCV13: 13-valent pneumococcal conjugate vaccine.

Table 2 shows pneumococcal vaccination coverages by underlying risk conditions. The highest PPsV23 coverage was observed among persons with congestive heart failure (81.6%) followed by chronic renal disease (77.4%), chronic bronchitis/emphysema (72.9%) and coronary artery disease (69.3%). Maximum PCV13 coverage was observed among patients with cirrhosis (3.7%) followed by chronic renal disease (3.5%), chronic bronchitis/emphysema (2.2%) and congestive heart failure (2.1%). The lowest PPsV23 coverage was observed among patients who abused alcohol (35.5%) and smoked (20.0%). Similarly, the lowest PCV13 coverage was also observed in people who smoke (0.5%) or abuse alcohol (0.7%).

**Table 2:** Pneumococcal vaccination coverages with PPsV23 and PCV13 by underlying risk conditions, Catalonia, Spain, 2017 (n = 2,057,656)

Risk condition/factor	PPsV23		PCV13	
	n	%	n	%
**Chronic respiratory diseases**				
Chronic bronchitis/emphysema (n = 132,306)	96,453	72.9	2,859	2.2
Asthma (n = 95,600)	52,817	55.2	1,218	1.3
**Chronic heart diseases**				
Congestive heart failure (n = 63,596)	51,909	81.6	1,327	2.1
Coronary artery disease (n = 117,575)	81,491	69.3	715	0.6
**Diabetes mellitus** (n = 350,511)	242,214	69.1	4,179	1.2
**Chronic renal disease** (n = 158,726)	122,791	77.4	5633	3.5
**Cirrhosis** (n = 7,957)	4,576	57.5	294	3.7
**Cancer** (n = 236,623)	139,911	59.1	3,758	1.6
**Alcoholism** (n = 64,829)	22,992	35.5	492	0.7
**Smoking** (n = 344,471)	68,966	20.0	1,646	0.5
**Splenectomy **(n = 362)	190	52.5	92	25.4

PPsV23: 23-valent pneumococcal polysaccharide vaccine; PCV13: 13-valent pneumococcal conjugate vaccine.

Table 3 shows distinct pneumococcal vaccine coverages (PPsV23, PCV13, dual vaccination, any vaccine) according to age subgroups (50–64 years, ≥ 65 years) and risk strata (healthy, at-risk and high-risk/immunocompromised) in the study population. Considering exclusively persons who had any type of risk condition, the proportion vaccinated with any vaccine was 16.1% (71,495/444,477) among those aged < 65 years and 76.4% (426,429/558,070) among those aged ≥ 65 years. Considering target groups where PPsV23 is recommended and publicly funded in Catalonia (i.e. all adults who are either < 65 years old with at least one risk factor plus all adults ≥ 65 years), PPsV23 coverage reached 52.8% (771,722/1,462,261). Considering exclusively persons for whom PCV13 is publicly funded in our setting (i.e. all-age immunocompromised/high-risk persons), PCV13 coverage was 3.3% (6,617/202,348).

**Table 3:** Pneumococcal vaccination coverages with PPsV23, PCV13, dual vaccination, or any vaccine, by age subgroups and risk strata among adults aged over 50 years, Catalonia, Spain, 2017 (n = 2,057,656)

Age groups	PPsV23		PCV13		PCV13 + PPsV23		PCV13 or PPsV23	
	n	%	n	%	n	%	n	%
**Aged 50–64** **years**								
Healthy (n = 595,395)	25,157	4.2	705	0.1	300	0.1	25,562	4.3
At-risk^a^ (n = 385,743)	58,318	15.1	1,097	0.3	615	0.2	58,800	15.2
High-risk^b^ (n = 58,734)	11,934	20.3	2,448	4.2	1,687	2.9	12,695	21.6
All (n = 1,039,872)	95,409	9.2	4,250	0.4	2,602	0.3	97,057	9.3
**Aged ≥ 65** **years**								
Healthy (n = 459,714)	275,700	60.0	1,607	0.3	1,371	0.3	275,936	60.0
At-risk^a^ (n = 414,456)	315,897	76.2	3,581	0.9	3,298	0.8	316,180	76.3
High-risk^b^ (n = 143,614)	109,873	76.5	4,169	2.9	3,793	2.6	110,249	76.8
All (n = 1,017,784)	701,470	68.9	9,357	0.9	8,462	0.8	702,365	69.0
**Overall (≥ 50** **years)**								
Healthy (n = 1,055,109)	300,857	28.5	2,312	0.2	1,671	0.2	301,498	28.6
At-risk^a^ (n = 800,199)	374,215	46.8	4,678	0.6	3,913	0.5	374,980	46.9
High-risk^b^ (n = 202,348)	121,807	60.2	6,617	3.3	5,480	2.7	122,944	60.8
All (n = 2,057,656)	796,879	38.7	13,607	0.7	11,064	0.5	799,422	38.9

PPsV23: 23-valent pneumococcal polysaccharide vaccine; PCV13: 13-valent pneumococcal conjugate vaccine.

^a^ Individuals considered at risk include immunocompetent persons with any of the following conditions: immunocompromising conditions, chronic pulmonary/respiratory diseases, heart diseases, chronic renal disease, diabetes mellitus, cirrhosis, alcoholism and/or smoking.

^b^ Individuals considered at high risk are immunocompromised persons.

Tables 4 and 5 show unadjusted and multivariable-adjusted analysis estimating ORs for PPsV23 and PCV13 vaccinations considering the distinct study covariables. In the multivariable analysis, age was the strongest predictor for PPsV23 vaccination. If we consider PCV13, the strongest predictor for vaccination was the presence of chronic renal disease (OR: 7.49; 95% CI: 7.19–7.79), followed by cirrhosis (OR: 4.21; 95% CI: 3.72–4.76) and chronic bronchitis/emphysema (OR: 2.54; 95% CI: 2.43–2.66).

**Table 4:** Unadjusted and multivariable-adjusted odds ratios for PPsV23 vaccination in adults, according to distinct study covariables, Catalonia, Spain, 2017 (n = 2,057,656)

Characteristic	Unadjusted			Multivariable-adjusted		
	OR	95% CI	p	OR	95% CI	p
**Age**						
50–64 years (reference)	1.00	Reference	< 0.001	1.00	Reference	< 0.001
65–79 years	16.91	16.77–17.05		14.21	14.09–14.34	
≥ 80 years	42.68	42.22–43.16		30.63	30.27–31.00	
**Sex**						
Male (reference)	1.00	Reference	< 0.001	1.00	Reference	< 0.001
Female	1.11	1.10–1.12		1.05	1.04–1.05	
**Chronic bronchitis/emphysema**	4.71	4.66–4.76	< 0.001	3.64	3.58–3.71	< 0.001
**Asthma**	2.02	1.99–2.05	< 0.001	2.21	2.17–2.25	< 0.001
**Congestive heart failure**	7.45	7.30–7.60	< 0.001	1.51	1.48–1.55	< 0.001
**Coronary artery disease**	3.87	3.82–3.92	< 0.001	1.61	1.59–1.64	< 0.001
**Diabetes mellitus**	4.64	4.61–4.68	< 0.001	3.74	3.70–3.78	< 0.001
**Cirrhosis**	6.21	6.13–6.29	< 0.001	1.81	1.70–1.92	< 0.001
**Chronic renal disease**	2.15	2.05–2.25	< 0.001	1.61	1.58–1.63	< 0.001
**Cancer**	2.56	2.54–2.59	< 0.001	1.32	1.31–1.34	< 0.001
**Alcoholism**	0.87	0.85–0.88	< 0.001	1.15	1.12–1.18	< 0.001
**Smoking**	0.34	0.33–0.34	< 0.001	0.68	0.67–0.69	< 0.001

CI: confidence interval; OR: odds ratio; PPsV23: 23-valent pneumococcal polysaccharide vaccine.

**Table 5:** Unadjusted and multivariable-adjusted odds ratios for PCV13 vaccination in adults, according to distinct study covariables, Catalonia, Spain, 2017 (n = 2,057,656)

Characteristic	Unadjusted			Multivariable-adjusted		
	OR	95% CI	p	OR	95% CI	p
**Age**						
50–64 years	1.00	Reference	< 0.001	1.00	Reference	< 0.001
65–79 years	2.14	2.06–2.23		1.11	1.06–1.16	
≥ 80 years	2.51	2.40–2.63		0.68	0.64–0.72	
**Sex**						
Male	1.00	Reference	< 0.001	1.00	Reference	< 0.001
Female	0.70	0.68–0.73		0.81	0.78–0.84	
**Chronic bronchitis/emphysema**	3.93	3.77–4.10	< 0.001	2.54	2.43–2.66	< 0.001
**Asthma**	2.03	1.91–2.16	< 0.001	1.83	1.72–1.94	< 0.001
**Congestive heart failure**	3.44	3.25–3.64	< 0.001	1.19	1.12–1.27	< 0.001
**Coronary artery disease**	2.40	2.28–2.53	< 0.001	1.14	1.08–1.21	< 0.001
**Diabetes mellitus**	2.17	2.10–2.25	< 0.001	1.30	1.25–1.35	< 0.001
**Cirrhosis**	8.73	8.43–9.03	< 0.001	4.21	3.72–4.76	< 0.001
**Chronic renal disease**	5.87	5.21–6.60	< 0.001	7.49	7.19–7.79	< 0.001
**Cancer**	2.97	2.86–3.08	< 0.001	2.13	2.04–2.21	< 0.001
**Alcoholism**	1.15	1.06–1.26	0.002	0.91	0.83–1.01	0.059
**Smoking**	0.68	0.65–0.72	< 0.001	0.80	0.76–0.85	< 0.001

CI: confidence interval; OR: odds ratio; PCV13: 13-valent pneumococcal conjugate vaccine.

## Discussion

Pneumococcal vaccination (including both PPsV23 and PCV13) is commonly recommended for individuals at risk or at high risk and older adults [2,3]. However, there is scarce available data about pneumococcal vaccination coverages among adults in recent years (after PCV13 licensure) and information on pneumococcal vaccination uptake is not routinely available.

The present large population-based study, involving more than 2 million persons ≥ 50 years old in Catalonia, shows an overall vaccination uptake of approximately 39% for the classical PPsV23 and 1% for the new PCV13 in January 2017. As compared with a prior study that evaluated vaccination coverages in Catalonia in January 2015 [7], current PPsV23 uptake is essentially similar (38.8% (789,098/2,033,465) in 2015 vs 38.7% (796,879/2,057,657) in 2017, p = 0.105) whereas PCV13 uptake, while very low, has slightly increased (from 0.2% (5,031/2,033,465) in 2015 to 0.7% (13,607/2,057,657) in 2017, p < 0.001). 

Our global pneumococcal vaccination coverage seems to be so far from the Healthy People 2020 goals (60% for at-risk adults aged < 65 years and 90% for elderly people) [4], but it must be noted that this global vaccination coverage is estimated considering the total population aged ≥ 50 years. Coverage rates are considerably greater when younger persons in this population (i.e. aged < 65 years) without underlying risk conditions are excluded in the analyses. Indeed, some specific population subgroups in this study have PPsV23 uptakes near to the Healthy People 2020 goal. If we consider exclusively elderly people, (i.e. ≥ 65 years), the observed PPsV23 coverage was 63.1% among persons 65–79 years and reached 81.2% among persons ≥ 80 years old. Similarly, high PPsV23 coverage emerged among persons with cardiac diseases (81.6% in congestive heart failure and 69.3% in coronary artery disease), chronic renal disease (77.4%) and chronic bronchitis/emphysema (72.9%).

If we consider PCV13, the global vaccine uptake remains very low in our setting at the beginning of 2017, despite being approved for use in adults since 2012 [3]. The lack of public funding in our setting (funded only for immunocompromised persons), together with its greater price and lower serotype-coverage than the classical PPsV23, could explain, in part, the very low PCV13 uptakes observed in our population.

Since pneumococcus is a major cause of morbidity and mortality in high-risk adults and elderly people, routine vaccination against pneumococcal diseases is recommended for these persons in many countries (although national guidelines differ, recommending either the PPsV23, the PCV13 or both). The classical PPsV23 offers 40–60% efficacy against invasive pneumococcal disease but its clinical effectiveness against pneumonia is uncertain [2]. The new PCV13 has better immunogenicity than the polysaccharide vaccine and has a demonstrated 45% effectiveness against vaccine-type pneumococcal pneumonia, but it has lower serotype-coverage than the PPsV23 [3,19]. The lowest vaccination coverages for both PPsV23 and PCV13 in this study were observed among people who smoked and people who abused alcohol. Both are at-increased risk to suffer pneumococcal infections and, consequently, vaccination uptakes should be largely improved in these groups. We underline the importance to continue public funding, especially among such target groups, to achieve reasonable vaccination uptakes. Barriers which limit vaccination uptake in these at-risk persons also require further investigation. In general, major factors related with vaccination acceptance in adults are self-perceived health and attitudes (which could be an important barrier in people who abuse alcohol and/or smoke) as well as perceptions about mortality risk of the infectious disease and vaccine effectiveness [20]. We underline the key role of general practice doctors in recommending vaccination in these persons [21].

In our multivariable analyses, age was the strongest predictor for PPsV23 vaccination, whereas the presence of some underlying conditions (particularly chronic renal disease, cirrhosis and chronic bronchitis/emphysema) was more predictive for PCV13 vaccination. These data fit with current pneumococcal vaccine recommendations in Catalonia, where PPSV23 is recommended (and publicly funded) for all persons aged ≥ 65 years (with or without underlying conditions) whereas PCV13 is only publicly funded for persons with high-risk conditions [9].

In Europe, there is very scarce population-based data reporting pneumococcal vaccination coverages among adults [7,21-26], and vaccine uptakes are generally inferred from vaccine doses distributed [22]. According to available data, estimates of PPsV23 uptakes among older adults in Western European countries vary largely (between 8% and 69%), with greater uptakes in those countries with age-based recommendations and public funding/reimbursement [21-26].

Considering that PCV13 use for at-risk and older adults was approved a few years ago (2012), there is very scarce published data about PCV13 uptakes in adult populations [6,7]. Our data showing a global PCV13 uptake less than one per cent (with maximum coverage of 3.7% in liver cirrhosis) 5 years after its licensure, suggests that, apart from public funding/reimbursement, risk-based vaccination strategies likely result in less vaccine uptake than age-based vaccination strategies [22]. In the US, where PCV13 is recommended for all persons aged ≥ 65 years (with or without risk conditions) [27], a coverage of 31% has been recently reported [6].

Considering the possibility of a sequential use of PCV13 plus PPsV23 (as recommended by the US Centers for Disease Control and Prevention for immunocompromised/high-risk persons and elderly people) [3,27], only 2.7% of high-risk individuals (5,480/202,348) had received dual PCV13/PPsV23 vaccination in our setting. To date, recommendations for PPsV23 and PCV13 vaccination in adults are not uniform (varying between countries, scientific societies and clinical guidelines), with diversity in funding for PPsV23/PCV13 in distinct settings, which makes it difficult to achieve optimal vaccine uptakes and limits the comparability of coverage rates [9,10,27-30].

Considering PPsV23, we also note the difficulty to improve vaccination uptakes when the vaccine coverage is already relatively high. In fact, the reported PPsV23 uptakes among elderly individuals and specific comorbidity subgroups in Catalonia years ago were similar to PPsV23 uptakes observed currently [31].

Our study has several strengths. Design was population-based and study population (which included more than 75% of the total Catalonian inhabitants ≥ 50 years) was representative and large enough to estimate accurately vaccination uptakes for both PPSV23 and PCV13 according to distinct age subgroups and specific underlying risk conditions. Quality criteria and utility of the SIDIAP research database (used to identify vaccinations, comorbidities and underlying risk conditions in the study population) have been previously reported [15], and this database has been used as a reliable tool for epidemiological studies in our setting [7].

With regard to limitations of the study, we did not include people < 50 years old in our analyses. Moreover, for people aged ≥ 50 years, we assumed that information in PCCs medical records was complete, but information bias may have occurred if some comorbidities and/or vaccinations were not recorded. Concerning vaccination, while the utility of the SIDIAP database for clinical research has been previously described [15], there has not been any validation sub-study specifically focused on vaccination registries in the database records. However possible bias/misclassification would likely be very small since almost all of adult vaccinations are administered in the PCCs in the Spanish Health System. Similarly, although it is possible that some comorbidities/chronic diseases (particularly in persons with mild symptoms or less frequency of attendance) were missed because a diagnosis code was not recorded, the possible misclassification would likely also be small considering that the observed prevalences fit with commonly reported prevalences for major comorbidities/risk conditions [32]. Patients attitudes, ethnicity and socio-economical information were not available in the present study, which may also be considered as a limitation given that disparities concerning these characteristics may influence pneumococcal vaccination coverage according to previous studies [20,33].

In conclusion, the present large population-based study shows that approximately 39% of overall Catalonian people aged ≥ 50 years (with or without risk conditions) had received at least one dose of the classical PPsV23 as at January 2017, whereas less than 1% had received the new PCV13. By population subgroups, PPsV23 uptakes were higher in older persons (reaching 81% among persons aged ≥ 80 years) and some subgroups with specific-comorbidities (81%, 77% and 73% among persons with cardiac, renal or pulmonary diseases, respectively). In contrast, considering PCV13, maximum uptakes did not even reach 4% (even considering certain at-risk population subgroups).

We highlight the difficulties to compare pneumococcal vaccination coverages in distinct countries due to the different characteristics of each national health service. Nevertheless, our study may provide valuable information for the evolution of pneumococcal vaccination coverage in a European region where PPsV23 is publicly funded for at-risk and high-risk adults and all elderly people whereas PCV13 is only publicly funded for high-risk individuals.

Recommendations for pneumococcal vaccination are currently heterogeneous depending on the distinct experts/clinical guidelines. To facilitate clinical practice and likely improve current vaccination coverage, more uniform recommendations (together with publicly funded programmes) for PPsV23/PCV13 use would be needed. These should be age-based, risk-based or consensus-based and derived from updated data on efficacy, clinical effectiveness, epidemiological impact and cost-effectiveness for both PPsV23/PCV13 in adults.
